# Burnout, Associated Factors, and Mental Health Measures Among Ecuadorian Physicians: A Cross-Sectional Study

**DOI:** 10.3390/jcm14072465

**Published:** 2025-04-04

**Authors:** Marina R. Ramírez, Mercy P. Ontaneda, Patricia Otero, David Ortega-Jiménez, Vanessa Blanco, Fernando L. Vázquez

**Affiliations:** 1 Department of Psychology, Universidad Técnica Particular de Loja, Loja 110107, Ecuador; mpontaneda@utpl.edu.ec (M.P.O.); dmortega1@utpl.edu.ec (D.O.-J.); 2 Department of Psychology, University of A Coruña, 15071 A Coruña, Spain; patricia.otero.otero@udc.es; 3 Department of Evolutionary and Educational Psychology, University of Santiago de Compostela, 15782 Santiago de Compostela, Spain; vanessa.blanco@usc.es; 4 Department of Clinical Psychology and Psychobiology, University of Santiago de Compostela, 15782 Santiago de Compostela, Spain; fernandolino.vazquez@usc.es

**Keywords:** burnout, correlates, psychological symptoms, physicians, health professionals

## Abstract

**Background**: Physician burnout is a growing issue that affects both the quality of healthcare and the mental well-being of medical professionals. However, research on this phenomenon in Ecuador is limited and methodologically deficient. The objective of this study was to examine the national prevalence of burnout among Ecuadorian physicians, its associated factors, and its impact on mental health. **Methods**: A cross-sectional study was conducted on 1976 physicians from all provincial capitals of Ecuador (51.8% women; mean age: 37.1 years). **Results**: It was found that 25.3% of physicians had high levels of emotional exhaustion and 23.8% had high levels of depersonalization. Factors associated with the higher levels of emotional exhaustion among physicians included part-time jobs, long working hours, work–family conflicts, psychological inflexibility, and perceived loneliness. Higher levels of depersonalization were associated with working shifts, having work–family conflicts, exhibiting psychological inflexibility, and perceived loneliness, while lower levels of depersonalization were associated with being female. Experiencing higher levels of depersonalization were associated with suffering from higher levels of depression, while having higher levels of emotional exhaustion were associated with suffering from higher levels of stress. **Conclusions**: A considerable proportion of Ecuadorian physicians suffer from burnout. Preventive programs and psychological interventions tailored to their specific needs should be developed.

## 1. Introduction

When work environments are not organized and managed well, they can have adverse consequences for workers that, rather than dignifying them, exhaust and consume their psychological resources [1]. Burnout constitutes an important psychosocial occupational risk in today’s society [1]. It is characterized as an inadequate response to chronic work-related stress that comprises emotional exhaustion, depersonalization, and reduced personal accomplishment [2]. According to Maslach and Jackson [2], emotional exhaustion refers to the feeling among workers that their ability to give to others on a psychological level is diminished as a result of their emotional resources being depleted. Depersonalization consists of the development of negative, cynical attitudes and feelings about one’s clients, or even a dehumanized perception of the clients, considering them as deserving of their troubles. Reduced personal accomplishment refers to the tendency to evaluate oneself negatively, particularly in relation to one’s work with clients, as well as feeling unhappy about oneself and dissatisfied with accomplishments in their job. However, previous research supports the idea that emotional exhaustion and depersonalization are the core dimensions of burnout, as they are more strongly related to job demands, stress, and mental health outcomes. Meanwhile, personal accomplishment develops independently and had weak correlations with these variables; it is conceptually distinct and more related to self-efficacy and work motivation [3,4].

Although burnout can occur in any profession, healthcare professionals have the highest prevalence of burnout [5]. Moreover, particularly among physicians, it has more serious implications for patients. It can undermine the medical care they receive, affect decision-making about their health, and cause medical errors that can have serious consequences [6].

According to the job demands–resources model [7,8], burnout occurs when job demands are high and job resources are low. Job demands refer to a job’s aspects (physical, psychological, or organizational) that require physical or mental effort, and incur costs for workers [8]. Job resources refer to the job aspects (physical, psychological, or organizational) that help reduce job demands and costs, help employees achieve professional goals, or promote their personal development [8]. In terms of job demands, certain sociodemographic and organizational variables can be risk factors for burnout in physicians and health workers, such as being a woman [9], being younger than 40 years of age, working in an urban area, having fewer than 10 years of professional experience, working shifts, working a greater number of hours, and attending to patients at risk of death [10,11,12,13,14,15,16]. Furthermore, despite the limited number of studies, scholars have also referred to psychosocial variables such as work–family conflicts [11,17], psychological inflexibility [18], and loneliness [19]. Meanwhile, job resources, marital status [20], a higher monthly income [11], greater professional experience, and social support [14,19] are related to lower levels of burnout among physicians.

The way these job demand factors affect burnout varies. For example, women, who are usually more emotional, experience burnout in a different way than men, who often report lower levels of depersonalization [21]. Younger professionals could be at higher risk for burnout due to not yet having the maturity and emotional control strategies that others gain over time [22]. Urban physicians have more job demands and exhaustion, which is associated with living and working in large cities [15]. Long hours and shift work compromise the sleep and rest needed by the professionals [23]. Attending patients at risk of death involves working under pressure and can result in work overload [16]. Additionally, work–family conflicts can consume the time and energy of professionals who are trying to combine work and family, increasing their work pressure and concerns and reducing job satisfaction, which can increase the likelihood of burnout [24]. Lastly, psychological inflexibility could reduce the availability of alternative solutions and reduce the resilience against adverse events [25]. Regarding job resources, marital status can also influence the development of burnout because professionals with partners can meet personal goals and strengthen other areas of their life beyond work, providing them with motivation to cope with their work [20]. The public sector increases autonomy and job security, and permanent contracts can reduce uncertainty and increase job satisfaction [26]. The level of income can influence the social image and job satisfaction of professionals [27]. The number of years of experience and a full-time appointment can increase professional skills and the salary achieved [28]. Social support provides instrumental and emotional assistance that provides resources and can act as a buffer for mental health issues [29].

Once burnout develops, it has important consequences for the person’s quality of life and health [30]. Burnout can be related to mental health problems such as depression, anxiety, and stress [31,32]. It also results in poor attention, an inability to concentrate, difficulty retaining information, recurring headaches, reduced sleep, feelings of fatigue, insecurity, helplessness, and poor work performance [33].

Since the onset of the COVID-19 pandemic, medical personnel have been exposed to considerable psychological pressure and excessive workloads, which dramatically increased the prevalence of burnout [34]. In Latin America, where healthcare systems have fewer resources, various studies (e.g., [35,36,37]) have reported that a third to more than half of physicians experienced burnout at some point during the pandemic. This, in turn, negatively impacted psychosocial health, resulting in distress and emotional overload [38]. Furthermore, it was associated with job dissatisfaction, absenteeism, and physician desertion [39,40]. Therefore, to improve healthcare system policies, it is critical to understand the predictors of burnout in medical professionals and the consequences for their mental health.

However, the existing research has some limitations. Most studies were conducted in North America or Europe (e.g., [12,13,17,18,19,41]). These studies considered overall dichotomized burnout as a dependent variable rather than a sum of its subscales (e.g., [13,17,31,42]). Few studies focused on core burnout subscales (emotional exhaustion and depersonalization) [12,13], while most used convenience samples [10,11,13,19,41], small sample sizes [13,19,41], and had low response rates [11,17,19,41]. Only two studies [43,44] have been conducted in Ecuador, despite it being an emerging country with the eighth largest economy in Latin America [45] and having a growing healthcare system with 23.2 physicians for every 10,000 inhabitants [46]. Among these studies, only the work of Ramírez et al. [43] was conducted at a national level. However, the authors evaluated physicians and nurses and did not present disaggregated results for each profession; therefore, the prevalence and correlates of burnout in physicians remain unknown. Meanwhile, Vinueza-Veloz [44] used a non-probabilistic sampling method, had a small sample, and did not include professionals working in the private health system. Moreover, the author only analyzed sex, age, profession (physician versus nurse), and level of care as potential correlates, and did not consider other relevant sociodemographic, work-related, or psychological variables. To the best of our knowledge, none of these studies focused solely on medical professionals or evaluated the psychological consequences of burnout. Thus, the national-level prevalence of burnout, factors associated with burnout, and variables related to mental health among Ecuadorian physicians are unknown.

To address these gaps, this study sought to determine the national prevalence of burnout (focusing solely on emotional exhaustion and depersonalization subscales), its correlates, and its consequences on the mental health of physicians in Ecuador.

## 2. Materials and Methods

### 2.1. Participants

A cross-sectional study was conducted. The sample comprised 1977 physicians from Ecuador, a South American country with an area of 256,370 km^2^ and 16,938,986 inhabitants [47]. The sample included first- (perform promotional, preventive, and palliative care functions), second- (perform specialized outpatient functions that require hospitalization), and third-level physicians (perform specialized outpatient and hospital activities). Participants were recruited from 79 public and private hospital centers in the capitals of the country’s 24 provinces from April to August 2022 using a stratified two-stage cluster sampling method. The first stage was based on territorial organization and included all provincial capitals. These capitals are distributed across coastal, highland, Amazonian, and insular regions, and have varying population densities ranging from sparsely populated and more rural areas (e.g., Puerto Baquerizo Moreno: 7290 inhabitants, with a population density of 3 inhabitants per km^2^) to more densely populated and urban areas (e.g., Guayaquil: 2,650,288 inhabitants, with a population density of 324 inhabitants per km^2^) [47]. Thus, we were able to capture wide heterogeneity in the study variables.

In the second stage, out of a total list of 4136 health centers in the Ecuadorian Health System, according to data from the Ministry of Public Health of Ecuador [48], 138 centers in the provincial capitals were randomly selected in proportion to the percentage of existing health centers in the different capitals. Specifically, approximately 48% of the health centers are concentrated in the most densely populated regions, 41% in intermediate population density regions, and 11% in low population density regions. Furthermore, to ensure the representativeness of the different types of health centers, the list of centers in each province was proportionally stratified by funding (public or private) and type of center (ambulatory health centers, basic and general hospitals, and specialized hospitals) through random sampling. As stated by the Ministry of Public Health of Ecuador [48], in terms of funding, 75% were public centers and 25% were private centers (which employ approximately 68% and 32% of the total number of physicians, respectively). Within this, in terms of the type of center, among public centers, 88.6% were ambulatory centers, 10% were general hospitals, and 1.4% were specialized hospitals. Meanwhile, among private centers, 29% were private ambulatory centers, 66% were general hospitals, and 5% were specialized hospitals. In each randomly selected health center, all the physicians were invited to participate in the study (through collaboration with the directors of the institutions who disseminated the study), which ensured that all those eligible had the same opportunity to be part of the study, minimizing the selection bias.

The sample size was calculated based on an estimated prevalence of burnout of 10%, as determined from a previous pilot study with a sample of 100 individuals from six health institutions (three private and three public) in the city of Loja, Ecuador (precision: ±2%; alpha error: 5%; expected sample loss: 20%).

The inclusion criteria required that subjects had to (a) be a physician, (b) be active, (c) have a minimum of one year of professional experience, and (d) provide informed consent. Those who were on sick leave or absent from their jobs for various reasons (e.g., vacations, stays, and training courses) at the time of the evaluation, as well as those who did not complete or did not correctly fill out the questionnaires, were excluded.

To minimize the loss of participants, the sample recruitment guidelines of Hulley et al. [49] were followed. These included treating participants with kindness and respect, presenting the study in an attractive way, helping them understand the research in a way that would make them want it to be successful, and collecting data in a pleasant manner and as least invasively as possible.

This study was conducted in accordance with the guidelines of the Declaration of Helsinki and was approved by the Human Research Ethics Committee of the Private Technical University of Loja, Ecuador, on 14 January 2022 (Cod. CEISH-03-2022). Informed consent was obtained from all participants, and the confidentiality of their responses was guaranteed. Participation was voluntary, and the participants did not receive any financial or other compensation.

### 2.2. Instruments

#### 2.2.1. Sociodemographic and Organizational Questionnaire

To evaluate the participants’ characteristics, an ad hoc questionnaire was used that included sociodemographic (sex, age, marital status, and area of work) and work-related variables (sector in which they work, type of contract, appointment, monthly income, professional experience, shifts, daily work hours, whether they treat patients at risk of death, and whether they are experiencing work–family conflicts). These variables were selected based on the job demands–resources model and clinical reasons.

#### 2.2.2. Burnout

To evaluate burnout, the Spanish version of the Maslach Burnout Inventory (MBI) [2] (Consulting Psychologists Press, Palo Alto, CA, USA) by Seisdedos [50] (Ediciones TEA, Madrid, Spain) was used. This self-administered instrument comprises 22 items with seven Likert-type response options ranging from 0 (never) to 6 (every day). It has three subscales: emotional exhaustion, depersonalization, and personal accomplishment. According to the scoring guidelines by Maslach et al. [2], for the emotional exhaustion subscale, a score >26 indicates a high level, a score between 19 and 26 indicates a medium level, and a score <19 indicates a low level. Regarding depersonalization, a score >9 indicates a high level, a score between 6 and 9 indicates a medium level, and a score <6 indicates a low level. For personal accomplishment, a score >39 indicates a high level, a score between 34 and 39 indicates a medium level, and a score <34 indicates a low level. The internal consistency (Cronbach’s alpha) of the original version of the subscales was 0.90 for emotional exhaustion, 0.79 for depersonalization, and 0.71 for personal accomplishment. For the purpose of this study and in agreement with previous research that has suggested that personal accomplishment measures a somewhat distinct dimension [3,4], burnout was considered as a two-dimensional construct composed of the continuous independent subscales of emotional exhaustion and depersonalization.

#### 2.2.3. Psychological Inflexibility

The Ecuadorian version of the Acceptance and Action Questionnaire (AAQ-II) [51] (London, UK) by Paladines-Costa et al. [52] (Loja, Ecuador) was used to evaluate psychological inflexibility. This self-administered instrument comprises seven items with seven Likert-type response options ranging from 1 (never) to 7 (always). It helps assess the unwillingness to experience unwanted emotions and thoughts, as well as the inability to be in the present moment and behave in accordance with value-directed actions when experiencing unwanted psychological events. Higher scores indicate higher levels of psychological inflexibility. The Cronbach’s alpha for this instrument was 0.92.

#### 2.2.4. Perceived Loneliness

To assess perceived loneliness, the Spanish version of the Three-Item Loneliness Scale [53] (Durham, NC, USA) by Trucharte et al. [54] (Madrid, Spain) was used. This is a brief three-item scale with three Likert-type response options ranging from 1 (almost never) to 3 (often). It evaluates the subjective feeling of unwanted loneliness, which is understood as the perception of having less social support than desired. Higher scores indicate greater loneliness. The Cronbach’s alpha for this instrument was 0.82.

#### 2.2.5. Symptoms of Depression, Anxiety, and Stress

To evaluate the symptoms of depression, anxiety, and stress, the Ecuadorian version of the Depression, Anxiety, and Stress Scale (DASS-21) [55] (Sydney, Australia) by Sanmartín et al. [56] (Quito, Ecuador) was used. It is a self-administered instrument made up of 21 items with four Likert-type response options ranging from 0 (not at all applicable to me) to 3 (very applicable to me), with three dimensions—depression, anxiety, and stress. Scores of ≥5 on depression, ≥3 on anxiety, and ≥7 on stress were considered indicative of their presence [57]. Overall, the DASS-21 presented a Cronbach’s alpha of 0.97, and the depression, anxiety, and stress subscales presented Cronbach’s alphas of 0.91, 0.91, and 0.90, respectively.

### 2.3. Procedure

A research protocol was developed to standardize the evaluation procedure, detailing the study objectives, design, and setting; participants (target population, accessible population, inclusion/exclusion criteria, sampling, and recruitment); measures (predictor and outcome variables); bias (non-response, recall bias, and selection bias); data analysis strategy; quality control; data management; schedule; and ethical issues.

Because two of the evaluation instruments (the MBI and Three-Item Loneliness Scale) have been previously validated in Spain but not in Ecuador, cultural adaptations were made to use them in a similar language (Spanish) but in another country (Ecuador) [58] according to the International Test Commission Guidelines [59]. Two researchers (native Ecuadorian Hispanophones familiar with Castilian Spanish) reviewed the battery of instruments, including the instructions, items, and response options. The researchers identified 17 terms in the MBI and 3 in the Three-Item Loneliness Scale that were unfamiliar to the Ecuadorian population and reworded them to Ecuadorian terms in an independent and parallel manner. Subsequently, an expert committee (comprising three experts in linguistics, and two researchers from the Spanish and Ecuadorian cultures) evaluated the semantic, idiomatic, experiential, and conceptual equivalence of the two versions of each instrument (Castilian and Ecuadorian) and resolved any flaws. The adaptations for these terms were of a lexical and linguistic nature and were made to retain the same meaning by referring to the same entities, maintaining the pragmatic meaning, and staying close to the lexical and structural features of the source text, without making any modification to the theoretical structure, the relationship between items, or the constructs measured. To ensure that our changes did not affect the structure of the instruments, we fully preserved the number of items, their order, and their response format, ensuring that the relationships between factors and indicators remained unchanged. Therefore, we did not modify the essence of the questions, nor did we introduce terms that would change their meaning. The modified versions were re-evaluated by the committee. The process was repeated until the committee reached a consensus on the equivalence of the two versions, and the pre-final version was determined. These versions were tested with Ecuadorian professionals and the results of these probes were reviewed by the expert committee, who made the final modifications and signed off on the final versions.

Subsequently, a pilot study was conducted to analyze feasibility using a sample of 100 individuals from six health institutions (three private and three public) in the city of Loja, Ecuador, who had characteristics similar to those who participated in the study. In order to support the stability of the psychometric properties after the terminological adaptation, a factor analysis was performed; it was found that the questionnaires retained the factor structure of the original studies, indicating that the linguistic changes did not impact the structural validity. In addition, the internal consistency of the instruments was evaluated using Cronbach’s alpha coefficient, obtaining values (0.91 for emotional exhaustion, 0.78 for depersonalization, 0.88 for personal accomplishment, and 0.88 for the Three-Item Loneliness Scale) similar to those reported in the original and Castilian Spanish versions. This shows that the items still measure the same constructs with an adequate level of reliability.

A total of 60 psychologists were trained to carry out the evaluations. The project was disseminated to psychology graduates nationwide from the administrative offices in charge of psychology degrees at the Private Technical University of Loja. The requirements were verified, acceptance was notified, and the training was conducted via Zoom, led by the members of the research project, over a period of 20 h. Subsequently, a letter was sent to the 138 health institutions in the 24 provinces presenting the objectives of the study and inviting them to participate. The collaboration of the directors of the institutions was sought through personal interviews to inform them of the research protocol and schedule dates for questionnaire collection; a total of 79 of them accepted. The application of the battery of evaluation instruments was performed online through the ArcGIS platform (Esri, version 2.8; Redlands, CA, USA). The participants were informed of the nature, objectives, risks, and benefits of the study. Confidentiality was guaranteed, and any concerns were answered. After obtaining informed consent, they accessed the battery of instruments to complete the evaluation. The average evaluation duration was 30–35 min.

### 2.4. Statistical Analysis

All data analyses were performed using the SPSS Statistical Package for Windows (IBM Corp., version 26; Armonk, NY, USA). For statistical or clinical reasons, some study variables were recoded. Following the recommendations of Dyrbye et al. [60], we reported the categorized results separately using established definitions of low, average, and high cut-off scores for each domain based on the scoring guidelines of Maslach et al. [2]. Other variables were dichotomized, i.e., marital status (without/with partner), shifts (no shifts/shifts), attend to patients at risk of death (no/yes), work–family conflicts (no/yes), depression (no/yes), anxiety (no/yes), and stress (no/yes). The means, standard deviations, and frequencies were calculated to analyze the distribution of the sociodemographic, organizational, and psychological variables of the sample, as well as the prevalence of burnout subscales (emotional exhaustion, depersonalization, and personal accomplishment).

We used linear regression to identify factors associated with the burnout subscales of emotional exhaustion and depersonalization. Similarly, linear regression analyses were performed to analyze the association between these subscales and reported symptoms of depression, anxiety, and stress in physicians. Prior to performing Poisson’s regression, the strength and direction of the relationships among the variables were determined using Pearson’s parametric correlation coefficient for two quantitative variables—biserial correlation coefficient for a quantitative and a dichotomous variable, and Cramer’s V for two dichotomous variables. Multicollinearity between variables was evaluated using the variance inflation factor (VIF) [61,62]. A value of 3 [63] was the threshold used as an indicator of multicollinearity. The strategy for combating multicollinearity was to sequentially eliminate the predictor with the highest VIF, recalculate the VIF for the rest of the variables, and repeat this process until all VIFs are smaller than the threshold of 3 [61,62]. All regression analyses were performed unadjusted and adjusted simultaneously for variables that in previous bivariate analyses reached *p*-values < 0.25, following Grant et al. [64], in addition to theoretical and clinical reasons for including the variables in the model.

## 3. Results

### 3.1. Sample Characteristics

**Figure A1:**
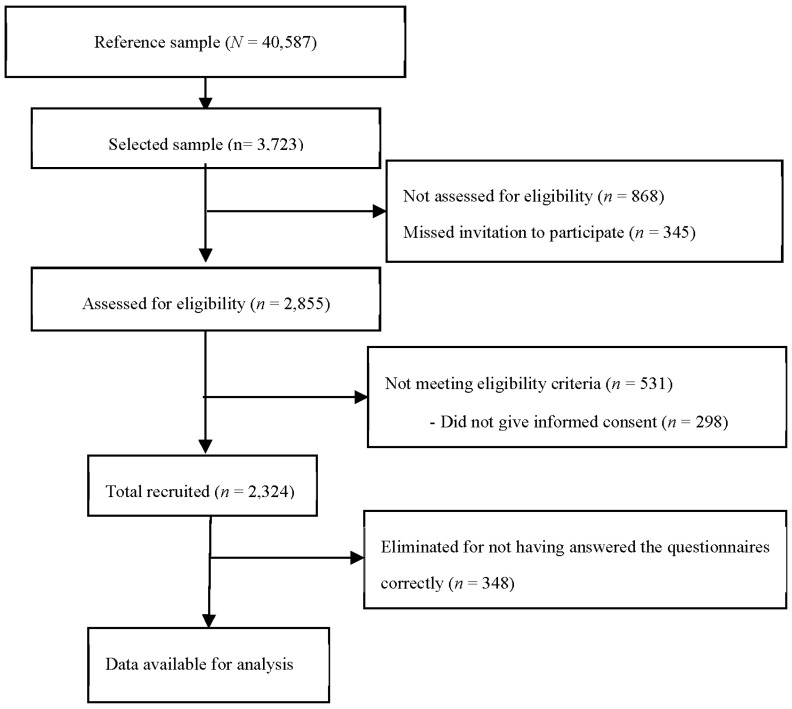
STROBE (Strengthening the Reporting of Observational Studies in Epidemiology) flow diagram.

The response rate was 86.0%. Of the total 3723 physicians selected, 523 (14.0%) refused to participate because of lack of time, busy schedules, lack of interest, or personal issues. No differences were observed between those who refused to participate and those who participated in terms of sociodemographic and job characteristics that we could discern (sex and sector in which they work). Among those who were assessed, 531 (14.3%) were excluded because they did not meet the eligibility criteria and 348 (9.3%) were eliminated because they did not answer the questionnaires correctly (see Appendix A Figure A1). A possible reason for this quantity of erroneously answered and incomplete questionnaires is that many physicians completed the questionnaires during their working hours, so they may not have had enough time and concentration to complete the questionnaires properly because they had to attend to a large number of patients during that time. The final sample comprised 1976 physicians (51.8% women; mean age: 37.1 years).

Women accounted for 51.8% of the participants, with a mean age of 37.1 years (*SD* = 9.4) (*M* = 38.3 for men and *M* = 36.0 for women). The majority of the participants did not have a partner (52.1%) (47.5% of men and 56.3% of women), worked in an urban area (84.7%) (85.7% of men and 83.7% of women), worked in the public sector (62.7%) (58.8% of men and 66.2% of women), had temporary contracts (56.1%) (52.6% of men and 59.3% of women), worked full time (83.8%) (85.0% of men and 82.6% of women), and had average incomes of USD 2828.1 per month (USD 2787.5 for men and USD 2865.7 for women). They had an average of 9.6 years (*SD* = 8.4) of work experience (10.4 for men and 8.9 for women), 59.4% worked shifts (61.4% of men and 57.5% of women), and worked an average of 10.5 h per day (*SD* = 5.4) (10.1 for men and 10.8 for women). A total of 85.6% cared for patients at risk of death (87.7% of men and 83.6% of women) and 66.7% experienced work–family conflicts (67.8% of men and 65.5% of women). The mean scores for psychological inflexibility and perceived loneliness were 16.6 (*SD* = 9.8) and 5.1 (*SD* = 2.3), respectively (16.9 for men vs. 16.4 for women, and 5.2 for men vs. 5.0 for women, respectively). Lastly, the mean scores for depression, anxiety, and stress were 3.6 (*SD* = 4.3), 3.9 (*SD* = 4.4), and 5.1 (*SD* = 4.6), respectively (3.6 for both men and women for depression, 3.8 for men vs. 4.0 for women for anxiety, and 4.9 for men vs. 5.2 for women for stress) (Table 1).

### 3.2. Prevalence of Burnout Syndrome

As shown in Table 2, the average score was 18.7 (*SD* = 11.7) for the emotional exhaustion subscale (*M* = 18.3 for men and *M* = 18.9 for women) and 6.0 (*SD* = 5.9) for depersonalization (*M* = 6.7 for men and *M* = 5.4 for women).

Notably, 25.3% (*n* = 500) had high levels of emotional exhaustion (24.7% of men and 25.9% of women) and 23.8% (*n* = 470) had high levels of depersonalization (27.1% of men and 20.7% of women).

### 3.3. Factors Associated with Burnout

Table 3 shows the correlation values between the variables. Some significant correlations were found, with associations ranging from weak (*r* = 0.05) to strong (*r* = 0.85). Subsequently, multicollinearity was corrected prior to the regression analysis by removing the variable with the highest VIF (age) from the analysis. As a result, all the variables included in the regression analysis had VIF values lower than 3.

As shown in Table 4 for the burnout syndrome subscales, a higher emotional exhaustion was significantly associated with working part time (β = 0.05; *p* = 0.005), working more hours per day (β = 0.04; *p* = 0.048), having work–family conflicts (β = 0.21; *p* < 0.001), exhibiting psychological inflexibility (β = 0.46; *p* < 0.001), and perceived loneliness (β = 0.11; *p* < 0.001). A lower depersonalization was significantly associated with being female (β = −0.13; *p* < 0.001), while a higher depersonalization was associated with working shifts (β = 0.11; *p* < 0.001), having work–family conflicts (β = 0.06; *p* = 0.001), exhibiting psychological inflexibility (β = 0.43; *p* < 0.001), and perceived loneliness (β = 0.12; *p* < 0.001). No significant differences were observed for the remaining variables.

### 3.4. Mental Health Symptoms in Physicians with and Without Burnout

As Table 5 shows, having higher levels of depersonalization were significantly associated with suffering from higher levels of depression (β = 0.07; *p* < 0.001). Meanwhile, experiencing high levels of emotional exhaustion was associated with suffering from higher levels of stress (β = 0.21; *p* < 0.001). No significant associations were found for the remaining variables.

## 4. Discussion

This study analyzed the prevalence of burnout subscales among physicians in Ecuador at the national level, as well as the factors and mental health symptoms associated with them. We found that 25.3% of physicians experienced high levels of emotional exhaustion (24.7% of men and 25.9% of women) and 23.8% experienced high levels of depersonalization (27.1% of men and 20.7% of women), indicating a high prevalence of burnout symptoms. These findings are consistent with those found in a review on physicians [65], where the prevalence was between 9.6% and 53.4% for emotional exhaustion, and between 11.0% and 47.7% for depersonalization. Our findings are slightly higher than the 17.2% and 13.5%, respectively, that were found for all healthcare professionals in Ecuador [43]. Additionally, our findings are in line with a previous study [19] that showed that burnout presents differently in men and women (e.g., a higher prevalence of emotional exhaustion in women and more depersonalization in men). However, these figures are concerning considering that our sample only included professionals who were working at the time of the study. In addition, one must consider that burnout symptoms could reduce the quality of care for patients [6] and result in suboptimal health outcomes.

Physicians who work part time, work more hours per day, have work–family conflicts, and exhibit psychological inflexibility and perceived loneliness were associated with higher emotional exhaustion. Those who work shifts, as well as having work–family conflicts, psychological inflexibility, and perceived loneliness, showed an association with higher levels of depersonalization, while women exhibited lower levels of depersonalization. Our finding that working part time was associated with a higher risk of depersonalization is consistent with a previous finding [14], which found that greater professional experience was related to less emotional exhaustion and depersonalization among physicians. An explanation for this could be that a full-time appointment can increase their professional skills, salary [26], and satisfaction, in addition to avoiding the precariousness and fatigue of having to work several jobs to earn a living. Our finding that working for working in shifts and for long hours increased the risk of emotional exhaustion and depersonalization for physicians was consistent with the evidence presented by the British Medical Association [66], as well as previous studies from around the world [10,14]. A possible explanation is that working under unfavorable conditions results in exhaustion and compromises the rest, leisure, and sleep of professionals. Specifically, shifts and long working hours can result in sleep deprivation and affect the physiological regulation of the circadian rhythm, in addition to pushing emotional and cognitive balances to their limit [67]. Further, similar to our work, previous studies found that work–family conflicts increase the risk of suffering high levels of emotional exhaustion and high levels of depersonalization [11,17]. This can be explained by the increase in the environmental demands for professionals and the resulting physical and emotional overload when they cannot cope or find a balance between their personal life and professional demands [24]. Psychological inflexibility was associated with experiencing a high level of emotional exhaustion and a high level of depersonalization. This is consistent with the findings of Jokic-Begic et al. [18], who found that greater psychological inflexibility in mental health professionals was related to greater levels of emotional exhaustion, depersonalization, and feelings of low personal accomplishment. A possible explanation is that the lack of skills to control one’s own unwanted emotions and thoughts is associated with worse psychological functioning during difficult times. Our finding that perceived loneliness was associated with a high level of emotional exhaustion and a high level of depersonalization is consistent with a previous study [14]. Wang et al. [14] found that workplace social support was negatively correlated with emotional exhaustion, depersonalization, and personal accomplishment. Possible explanations include the following: (1) the almost exclusive dedication to a professional career in medicine, which leaves little time to cultivate personal relationships; (2) the high demands and a climate of competitiveness starting from the stage of academic training; and (3) the strict hierarchy among healthcare workers, resulting in relationships that lack mutual support in the workplace. Thus, interpersonal relationships between healthcare colleagues are hostile, unsafe, and a source of tension [68].

Furthermore, females exhibited a negative association with high levels of depersonalization. This finding is consistent with that of Mijakoski et al. [12], who found a lower risk in female physicians, but only for the depersonalization dimension. Hence, a possible explanation may be that the women in the sample of Ecuadorian physicians presented greater empathy and vocation for caring, which increased their engagement in the performance of their profession.

No associations were found between the other variables analyzed and the burnout subscales. It is particularly surprising that professional experience had no association with burnout subscales, contradicting the established literature [14,43], which has found that greater experience is a protective factor, potentially due to their higher confidence surrounding their professional skills. This could be explained by the fact that in Ecuador, there is a complete and structured training program for young physicians in which they receive close support during the acquisition of professional skills, so that when they start their professional career they have already acquired some experience and professional skills. A medical degree takes place over six years, with the last year being a rotating internship in hospitals and health centers where students apply their knowledge in real-life settings under supervision. Subsequently, they must complete a year of mandatory rural medical service, where they must work in rural areas providing primary care. This is followed by the specialization program, which lasts three to five years depending on the specialty. In addition, there are continuing education programs for those seeking to update or specialize in specific areas. However, it may also be possible that our finding represents a cohort effect, reflecting the early departure from clinical practice by those staff members who eventually succumb to burnout.

Meanwhile, it was found that exhibiting higher levels of depersonalization was associated with higher levels of depression, while having higher levels of emotional exhaustion was associated with suffering from higher levels of stress. Nevertheless, these results should be taken with caution, as they only demonstrate association and not causality. These findings are partially consistent with a previous study [69], which found that healthcare workers with emotional exhaustion and disengagement had more symptoms of depression and anxiety compared to those without burnout. A possible explanation for this is the hypothesis of burnout as a “loss spiral” or “burnout cascade” [70]. This hypothesis postulates that the severity of the consequences of burnout is related to its course, which ranges from the loss of empathy to depression or anxiety.

Our findings have important implications for research, health policy, and clinical practice. They reveal that a considerable number of physicians in Ecuador are at the limit of their endurance at work. The work, personal factors, and the mental health problems associated with burnout subscales were identified in this study. Although no causal relationship between these variables could be determined, the association between them suggests that coordinated action at the individual, organizational, and policy-making levels could help medical professionals. For instance, the work structure characterized by rotating shifts and long hours (including shifts of 24 h or more) should be changed as it hinders the integration between work and personal life. Instead, a reduction in the working day, stable shifts, and work–life balance measures (especially in more demanding and sensitive periods, such as maternity/paternity, family illness, or caring for dependent family members) should be implemented. Additionally, the work culture, which is based on competitiveness, needs to be reviewed. Instead, efforts should be undertaken to promote teamwork and develop support networks in the work environment. Meanwhile, given the influence of psychological inflexibility on burnout subscales and the psychological symptoms associated with them, psychological support is needed to preserve the mental health of medical personnel, including training in psychological techniques that help them cope with negative emotions and thoughts. Specifically, mindfulness-based interventions, such as meditation or stress reduction programs, have demonstrated benefits for healthcare workers in terms of ameliorating burnout-related symptoms [71]. Programs that create a people-centered work culture can help balance positive and negative situations, enabling physicians to thrive [72].

This study has some limitations. The cross-sectional nature of the study did not allow us to establish causality between the variables, deduce the direction of the relationship between burnout subscales and mental health, or determine the influence of possible mediating variables on the analyzed relationships. Longitudinal studies are necessary to prospectively evaluate burnout symptoms and mental health problems, as well as potential moderating variables. Comparisons between studies are limited to studies with the same burnout conceptualization, since different classification methods may yield varying prevalence estimates. Although the correlation analysis provided relevant information about risk factors, future studies should conduct pathway analyses to explain how the various variables are interrelated. Additionally, this study employed self-reporting instruments, which could introduce a response bias. Moreover, because two of the instruments (the MBI and Three-Item Loneliness Scale) have been previously validated in Spain but not in Ecuador, cultural adaptations were made to use them in Ecuador, including rewording to Ecuadorian terms in the items that were unfamiliar to the Ecuadorian population. Lastly, data on the specialties of the physicians were not collected in this study, despite the fact that there are studies that point out that some specialties (such as surgeons) are exposed to high levels of burnout [73]. Future studies should address this issue. Nonetheless, this is the first study to estimate the prevalence of burnout subscales among physicians in Ecuador at the national level, as well as its association with mental health symptoms. The large sample size, high response rate, and representativeness of the sample at the national level support the generalizability of the results.

## 5. Conclusions

Our findings revealed that a considerable number of physicians in Ecuador exhibit emotional exhaustion and depersonalization.

Factors associated with the higher levels of emotional exhaustion of physicians were having a part-time job, long working hours, work–family conflicts, psychological inflexibility, and perceived loneliness. Higher levels of depersonalization were associated with working shifts, having work–family conflicts, exhibiting psychological inflexibility, and perceived loneliness, while lower levels of depersonalization were associated with women. Experiencing higher levels of depersonalization was associated with suffering from higher levels of depression, while having higher levels of emotional exhaustion was associated with suffering from higher levels of stress. The development of specific cognitive–behavioral interventions to prevent burnout symptoms should be a priority of future research and healthcare policies.

## Figures and Tables

**Table 1:** Characteristics of participants.

Characteristic	Total		Men		Women	
	*n* (%)	*M* (*SD*)	*n* (%)	*M* (*SD*)	*n* (%)	*M* (*SD*)
Sex Men Women	952 (48.2) 1024 (51.8)					
Age		37.1 (9.4)		38.3 (10.0)		36.0 (8.7)
Marital status Without partner With partner	1029 (52.1) 947 (47.9)		452 (47.5) 500 (52.5)		577 (56.3) 447 (43.7)	
Area of work Urban Rural	1673 (84.7) 303 (15.3)		816 (85.7) 136 (14.3)		857 (83.7) 167 (16.3)	
Sector in which they work Public Private/mixed	1238 (62.7) 738 (37.3)		560 (58.8) 392 (41.2)		678 (66.2) 346 (33.8)	
Type of contract Temporary Permanent	1108 (56.1) 868 (43.9)		501 (52.6) 451 (47.4)		607 (59.3) 417 (40.7)	
Appointment Part time Full time	321 (16.2) 1655 (83.8)		143 (15.0) 809 (85.0)		178 (17.4) 846 (82.6)	
Monthly income		2828.1 (3445.7)		2787.5 (3302.5)		2865.7 (3574.8)
Professional experience		9.6 (8.4)		10.4 (8.9)		8.9 (7.8)
Shifts No shifts With shifts	802 (40.6) 1174 (59.4)		367 (38.6) 585 (61.4)		435 (42.5) 589 (57.5)	
Daily workday		10.5 (5.4)		10.1 (5.0)		10.8 (5.7)
Attend to patients at risk of death No Yes	285 (14.4) 1691 (85.6)		117 (12.3) 835 (87.7)		168 (16.4) 856 (83.6)	
Work–family conflicts No Yes	658 (33.3) 1318 (66.7)		328 (34.5) 624 (65.5)		330 (32.2) 694 (67.8)	
Psychological inflexibility		16.6 (9.8)		16.4 (9.7)		16.9 (9.9)
Perceived loneliness		5.1 (2.3)		5.0 (2.3)		5.2 (2.3)
Depression		3.6 (4.3)		3.6 (4.5)		3.6 (4.1)
Anxiety		3.9 (4.4)		3.8 (4.4)		4.0 (4.3)
Stress		5.1 (4.6)		4.9 (4.6)		5.2 (4.5)

**Table 2:** Prevalence of burnout syndrome and its subscales in the participants.

Variable	Total	Sex	
	*n* (%)	Men *n* (%)	Women *n* (%)
Emotional Exhaustion Subscale *M* (*SD*) Low Medium High	18.7 (11.7) 1107 (56.0) 369 (18.7) 500 (25.3)	18.3 (12.0) 538 (56.5) 179 (18.8) 235 (24.7)	18.9 (11.4) 569 (55.6) 190 (18.6) 265 (25.9)
Depersonalization Subscale *M* (*SD*) Low Medium High	6.0 (5.9) 1245 (63.0) 261 (13.2) 470 (23.8)	6.7 (6.2) 553 (58.1) 141 (14.8) 258 (27.1)	5.4 (5.6) 692 (67.6) 120 (11.7) 212 (20.7)

**Table 3:** Correlations between all the variables.

	(1)	(2)	(3)	(4)	(5)	(6)	(7)	(8)	(9)	(10)	(11)	(12)	(13)	(14)	(15)	(16)	(17)	(18)	(19)	(20)
1. Emotional exhaustion	1																			
2. Depersonalization	0.63 **	1																		
3. Sex (0 = Men;1 = Women)	0.03	−0.11 **	1																	
4. Age	−0.18 **	−0.16 **	−0.12 **	1																
5. Marital status (0 = Without partner; 1 = With partner)	−0.13 **	−0.14	0.09 **	0.34 **	1															
6. Area of work (0 = Urban; 1 = Rural)	0.05 *	0.05 *	0.03	−0.16 **	0.06 *	1														
7. Sector in which they work (0 = Public; 1 = Private)	−0.04	0.01	0.08 *	0.11 **	0.02	0.19 **	1													
8. Type of contract (0 = Temporary; 1 = Permanent)	−0.07 **	−0.03	0.07 **	0.31 **	0.15 **	0.16 **	0.07 **	1												
9. Appointment (0 =Full time; 1 = Part time)	−0.06 **	−0.04	−0.03	−0.06 *	0.04	0.07 **	0.18 **	0.16 **	1											
10. Income	−0.02	−0.03	0.01	−0.03	−0.05 *	−0.70 **	0.15 **	−0.07 **	0.01	1										
11. Professional experience	−0.13 **	−0.13 **	−0.09 **	0.82 **	−0.10 **	−0.13 **	0.12 **	0.26 **	−0.04	−0.01	1									
12. Shifts (0 = No Shifts; 1 = With shifts)	0.05 *	0.15 **	0.04	−0.16 **	0.08 **	0.21 **	0.01	0.10 **	0.01	0.03	−0.15 **	1								
13. Daily workday	0.04	0.01	0.06 **	−0.17 **	0.27 **	−0.10 **	−0.15 **	0.05 *	−0.06 **	−0.03	−0.17 **	0.37 **	1							
14. Attend to patients at risk of death (0 = No; 1 = Yes)	0.08 **	0.05 *	0.06 **	−0.04	0.04	0.04	0.13 **	0.06 *	0.08 **	−0.04	−0.06 **	0.17 **	0.15 **	1						
15. Work–family conflicts	0.33 **	0.17 **	0.02	−0.03	0.01	0.03	0.01	0.05 *	0.04	−0.05 *	−0.03	0.01	−0.02	0.10 **	1					
16. Psychological inflexibility	0.58 **	0.53 **	0.02	−0.21 **	−0.18 **	0.05 *	0.01	−0.09 **	−0.01	0.01	−0.17 **	0.09 **	−0.01	0.08 **	0.20 **	1				
17. Perceived loneliness	0.49 **	0.41 **	0.03	−0.14 **	−0.19 **	0.07 **	−0.03	−0.04	−0.01	−0.01	−0.10 **	0.05 *	0.01	0.05 *	0.19 **	0.63 **	1			
18. Depression	0.55 **	0.53 **	−0.01	−0.17 **	−0.12 **	0.07 **	−0.03	−0.08 **	−0.02	0.01	−0.13 **	0.10 **	−0.01	0.07 **	0.18 **	0.70 **	0.55 **	1		
19. Anxiety	0.55 **	0.50 **	0.02	−0.14 **	−0.11 **	0.04	−0.01	−0.06 *	−0.02	0.01	−0.12 **	0.11 **	0.02	0.08 **	0.19 **	0.66 **	0.53 **	0.83 **	1	
20. Stress	0.64 **	0.53 **	0.04	−0.17 **	−0.11 **	0.05 *	−0.03	−0.05	−0.02	0.01	−0.13 **	0.09 **	0.01	0.08 **	0.23 **	0.68 **	0.54 **	0.83 **	0.85 **	1

Note: * *p* < 0.05; ** *p* < 0.01.

**Table 4:** Correlates of burnout subscales among physicians using linear regression.

Characteristic	Emotional Exhaustion					Depersonalization				
	β	Standard Error	*t*	*p*	95% CI ^a^	β	Standard Error	*t*	*p*	95% CI ^b^
Sex (0 = Men 1 = Women)	0.01	0.42	0.02	0.986	−0.81, 0.83	−0.13	0.22	−6.98	<0.001	−1.96, −1.10
Marital status (0 = Without partner 1 = With partner)	−0.03	0.43	−1.57	0.117	−1.51, 0.17	−0.04	0.23	−1.84	0.065	−0.88, 0.27
Area of work (0 = Urban 1 = Rural)	0.01	0.61	0.22	0.828	−1.10, 1.32	0.04	0.32	1.82	0.069	−0.05, 1.19
Sector in which they work (0 = Public 1 = Private)	−0.03	0.45	−1.62	0.105	−1.61, 0.15	0.01	0.23	0.58	0.563	−0.32, 0.59
Type of contract (0 = Temporary 1 = Permanent)	−0.02	0.43	−1.30	0.194	−1.41, 0.29	0.01	0.24	0.73	0.469	−0.29, 0.63
Appointment (0 = Full time 1 = Part time)	0.05	0.58	2.80	0.005	0.48, 2.74	−0.04	0.30	−1.84	0.065	−1.14, 0.04
Monthly income	−0.01	0.01	−0.30	0.768	−0.99, 1.01	−0.03	0.01	−1.52	0.128	−0.98, 1.01
Professional experience	−0.01	0.03	−0.68	0.496	−0.07, 0.04	−0.03	0.01	−1.59	0.113	−0.05, 0.01
Shifts (0 = No Shifts 1 = With shifts)	0.01	0.47	0.38	0.701	−1.10, 0.73	0.11	0.23	5.41	<0.001	0.81, 1.73
Daily workday	0.04	0.04	1.90	0.048	0.01, 0.16	0.04	0.02	1.81	0.070	−0.08, 0.03
Patients at risk of death (0 = No 1 = Yes)	0.01	0.61	0.63	0.530	−0.81, 1.57	−0.02	0.32	−1.05	0.292	−0.96, 0.29
Work–family conflict (0 = No 1 = Yes)	0.21	0.45	11.59	<0.001	4.32, 6.08	0.06	0.24	3.26	0.001	0.31, 1.24
Psychological inflexibility	0.46	0.03	20.07	<0.001	0.50, 0.61	0.43	0.02	17.51	<0.001	0.23, 0.28
Perceived loneliness	0.11	0.12	4.74	<0.001	0.32, 0.78	0.12	0.06	4.95	<0.001	0.18, 0.42

Notes: CI = confidence interval. ^a^ Adjusted for marital status, area of work, sector in which they work, type of contract, appointment, professional experience, shifts, daily workday, attendance to patients at risk of death, work–family conflicts, psychological inflexibility, and perceived loneliness. ^b^ Adjusted for sex, marital status, area of work, type of contract, appointment, professional experience, shifts, attendance to patients at risk of death, work–family conflicts, psychological inflexibility, and perceived loneliness.

**Table 5:** Mental health symptoms among physicians based on the burnout subscales using linear regression.

Characteristic		Depression					Anxiety					Stress			
	β	Standard Error	*t*	*p*	95% CI ^a^	β	Standard Error	*t*	*p*	95% CI ^b^	β	Standard Error	*t*	*p*	95% CI ^c^
Emotional Exhaustion	−0.01	0.01	−0.12	0.905	−0.01, 0.01	−0.02	0.01	−1.46	0.144	−0.02, 0.03	0.21	0.01	14.81	<0.001	0.07, 0.09
Depersonalization	0.07	0.01	4.84	<0.001	0.03, 0.08	0.02	0.01	1.07	0.284	−0.10, 0.03	−0.01	0.01	−0.05	0.957	−0.02, 0.02

Note: CI = confidence interval. ^a^ Adjusted for emotional exhaustion, depersonalization, anxiety, and stress. ^b^ Adjusted for emotional exhaustion, depersonalization, depression, and stress. ^c^ Adjusted for emotional exhaustion, depersonalization, depression, and anxiety.

## Data Availability

The data presented in this study are available on request from the corresponding author. The data are not publicly available due to confidentiality issues.
